# Apolipoprotein E is a marker of all chondrocytes in the growth plate resting zone

**DOI:** 10.1038/s41413-025-00407-2

**Published:** 2025-03-03

**Authors:** Joe Kodama, Takeshi Oichi, Kevin J. Wilkinson, Joshua M. Abzug, Takashi Kaito, Motomi Enomoto-Iwamoto, Masahiro Iwamoto, Satoru Otsuru

**Affiliations:** 1 Department of Orthopaedics, University of Maryland School of Medicine, Baltimore, MD USA; 2 Department of Orthopaedic Surgery, Osaka University Graduate School of Medicine, Osaka, Japan

**Keywords:** Physiology, Bone

## Abstract

The resting zone (RZ) in mammalian growth plates is critical for maintaining and regulating chondrocyte turnover during longitudinal bone growth as a control tower and stem cell reservoir. Although recent lineage tracing studies have identified several markers for stem cells in the RZ, these markers only partially label chondrocytes in the RZ, suggesting that the resting chondrocytes (RCs) are a heterogeneous population with different types of stem cells. Since a comprehensive marker for RCs is still lacking, the RZ is generally determined based on ambiguous histological criteria, such as small and round chondrocytes without columnar formation, which may lead to inconsistencies among researchers. Therefore, in this study, we used single-cell RNA sequencing (scRNAseq) of growth plate chondrocytes followed by validation by fluorescence in situ hybridization (FISH) to precisely annotate cell clusters in scRNAseq and search for a marker of RCs. The scRNAseq analysis revealed that apolipoprotein E (*Apoe*) was the top-hit gene, which was ubiquitously expressed in the RC cluster. FISH confirmed that *Apoe* was exclusively localized to the histologically defined RZ. In newly generated *Apoe*^*mCherry*^ knock-in mice, we further confirmed that mCherry expression mirrored the distribution of *Apoe*-expressing chondrocytes in the RZ particularly after the formation of the secondary ossification center. These mCherry^+^ RCs were slow cycling in vivo and exhibited stem cell properties in vitro. Moreover, APOE was detected in human growth plate RCs. These findings suggest that apolipoprotein E is a novel pan-RC marker in both mouse and human growth plates.

## Introduction

Postnatal longitudinal bone growth occurs by explosive endochondral bone formation in the cartilaginous growth plates located at both ends of long bones between the epiphysis and metaphysis. Histologically, despite the high cell turnover, the growth plate is well-organized and stratified into a resting zone (RZ), proliferative zone (PZ), prehypertrophic zone (PHZ), and hypertrophic zone (HZ). The RZ, which contains round resting chondrocytes (RCs), is situated at the top of the growth plate, closest to the secondary ossification center (SOC). The RCs then give rise to flattened amplifying chondrocytes, which stack in columns in the PZ. Subsequently, the chondrocytes exit the cell cycle and enter the maturing stage in the PHZ. The cells then increase in size to become mature hypertrophic chondrocytes and induce calcification of the surrounding extracellular matrix in the HZ. Eventually, these terminally differentiated hypertrophic chondrocytes undergo apoptosis or transdifferentiation into osteoblasts,^1–3^ exit the growth plate, and the remaining calcified cartilage is replaced by bone.^4,5^ This rapid and continuous turnover of the growth plate chondrocytes must be precisely regulated, especially during the growth spurt.^6–8^

In mice, after SOC formation, the RZ harbors skeletal stem cells that undergo self-renewal and asymmetric cell division to continuously supply chondrocytes to form columns in the proliferative zone.^9^ In addition to being a cellular reservoir, the RZ also plays a regulatory role in chondrocyte proliferation and maturation by secreting paracrine factors such as PTHrP.^10^ Moreover, in the vicinity of the vascularized SOC, the RCs act as sensors of systemic hormonal and nutrient signals that regulate linear growth.^6,8^

However, to date, there is no fully accepted criterion for molecularly defining the RZ and/or RCs. As a result, most of the studies investigating the RZ in growth plates use ambiguous definitions of RCs based on their cell shape, making it difficult to accurately separate the RZ from the PZ without interpersonal bias. Recent studies have identified several markers that label stem cells in the RZ, such as *Pthlh*,^11^ *Axin2*,^12,13^ and *Foxa2*.^14^ Lineage tracing of stem cells expressing these markers has successfully demonstrated that these stem cells can form columns in the growth plate. However, none of these markers could completely label all RCs or all the columns in the entire growth plate, suggesting that RCs are heterogeneous populations and that these markers may label subsets of RCs. Therefore, we sought to identify pan-RC markers that are commonly but specifically expressed in RCs by transcriptomic analysis of growth plate chondrocytes.

Single-cell RNA sequencing (scRNAseq) has become widely available. It is becoming indispensable to transcriptionally characterize cell populations at a single-cell resolution and to investigate the molecular mechanisms of disease. However, scRNAseq transcriptomics loses the spatial information of the cells during tissue digestion to prepare single-cell suspensions, which can make it difficult to characterize cells of interest identified histologically. On the other hand, spatial transcriptomics of hard tissues is still technically challenging, and single-cell resolution has not been achieved.^15,16^ Therefore, in this study, we utilized RNA fluorescence in-situ hybridization (FISH, RNAscope) with comprehensive gene panels on mouse growth plate tissues to spatially identify and accurately annotate the cell clusters identified by scRNAseq analysis of growth plate chondrocytes. This approach allowed us to distinguish RCs from other cell types and identify transcriptomes that specifically characterize RC.

## Results

### scRNAseq analysis and FISH validation precisely identified clusters of the growth plate chondrocytes

**Fig. 1:**
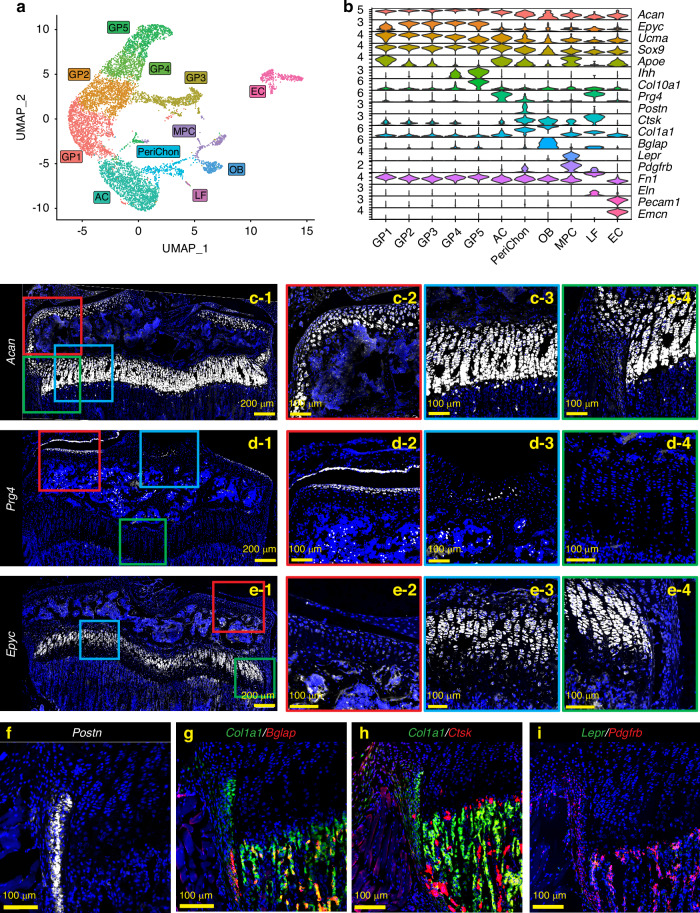
scRNAseq analysis and FISH validation of the single cells isolated from hindlimb growth plates. **a** UMAP analysis of single cells obtained from the digested hindlimb epiphyseal growth plates of a 4-week-old female C57BL/6 J mouse based on the transcriptomic profiles of each cell. **b** Violin plots of the gene panel selected for cluster annotation and histological validation. **c**–**i** FISH validations for representative genes for cluster annotation. **c-2**-**4**, **d-2**-**4** and **e-2**-**4** are magnified images of the corresponding colored boxes indicated in **c-1**, **d-1**, and **e-1**. At least 3 mice were used for each gene and the representative images were shown

**Fig. 2:**
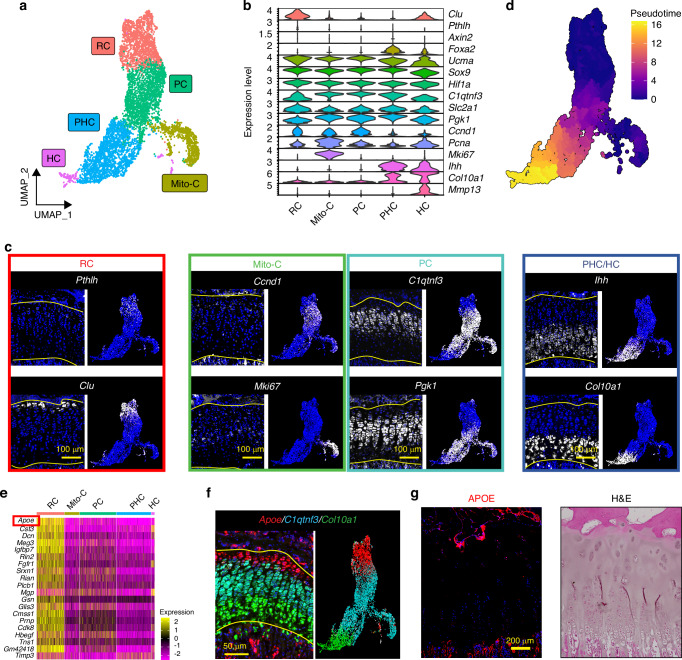
Characterization of RCs by scRNAseq and FISH to identify markers for RCs. **a** Reclustering and UMAP visualization of the GP chondrocytes selected in Fig.1a. **b** Gene panel used for cluster annotation of GP chondrocytes and FISH validation. **c** FISH images and the corresponding feature plots of genes representing chondrocytes in different zones. **d** Pseudotime analysis of the GP chondrocytes. Higher pseudotime scores indicate more mature/differentiated chondrocytes. **e** Heatmap of the detected genes that are ubiquitously and specifically expressed in the RC cluster. **f** FISH for *Apoe*, *C1qtnf3*, and *Col10a1* on a coronal section of a 4-week-old male mouse tibia. Feature plot of the three genes is shown in corresponding colors next to the FISH image. **g** Hematoxylin & Eosin (H&E) staining and immunofluorescence staining of human APOE on sections of the human radial growth plate. At least 3 mice were used for each gene and the representative images were shown

**Fig. 3:**
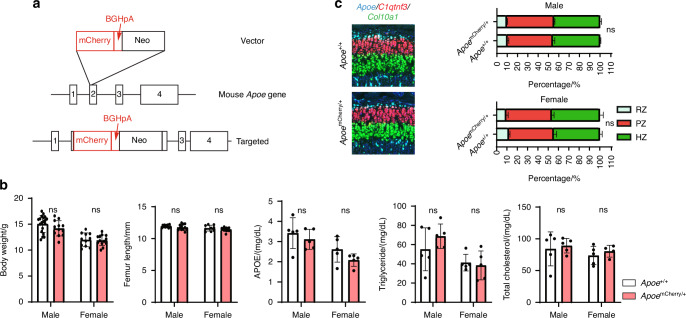
Systemic and skeletal phenotypes of newly generated *Apoe*^*mCherry/+*^ mice. **a** Gene editing strategy to generate *Apoe*^*mCherry*^ knock-in mice. **b** Body weights (*Apoe*^*+/+*^ males = 19, *Apoe*^*+/+*^ females = 11, *Apoe*^*mCherry/+*^ males = 12, *Apoe*^*mCherry/+*^ females = 13), femur lengths measured by micro-CT scanning (*Apoe*^*+/+*^ males = 11, *Apoe*^*+/+*^ females = 9, *Apoe*^*mCherry/+*^ males = 12, *Apoe*^*mCherry/+*^ females = 11), plasma APOE levels measured by ELISA (*n* = 5 for each group), and blood lipid levels measured by colorimetric assays (*n* = 5 for each group except the total cholesterol level in the *Apoe*^*mCherry/+*^ group which is *n* = 4). Samples were from independent animals. Data were presented as mean ± SD. Two-way ANOVA followed by Tukey’s multiple comparison test. **c** Tibial sections from 4-week-old male *Apoe*^*+/+*^ and *Apoe*^*mCherry/+*^ mice (*n* = 3 independent animals) were stained by FISH for *Apoe*, *C1qtnf3*, and *Col10a1* to label the RZ, PZ, and HZ, respectively. Percentage of each zone within the entire growth plate was quantified and compared by Two-way ANOVA followed by Tukey’s multiple comparisons test. Data are presented as mean ± SD. ns: *P* > 0.05

**Fig. 4:**
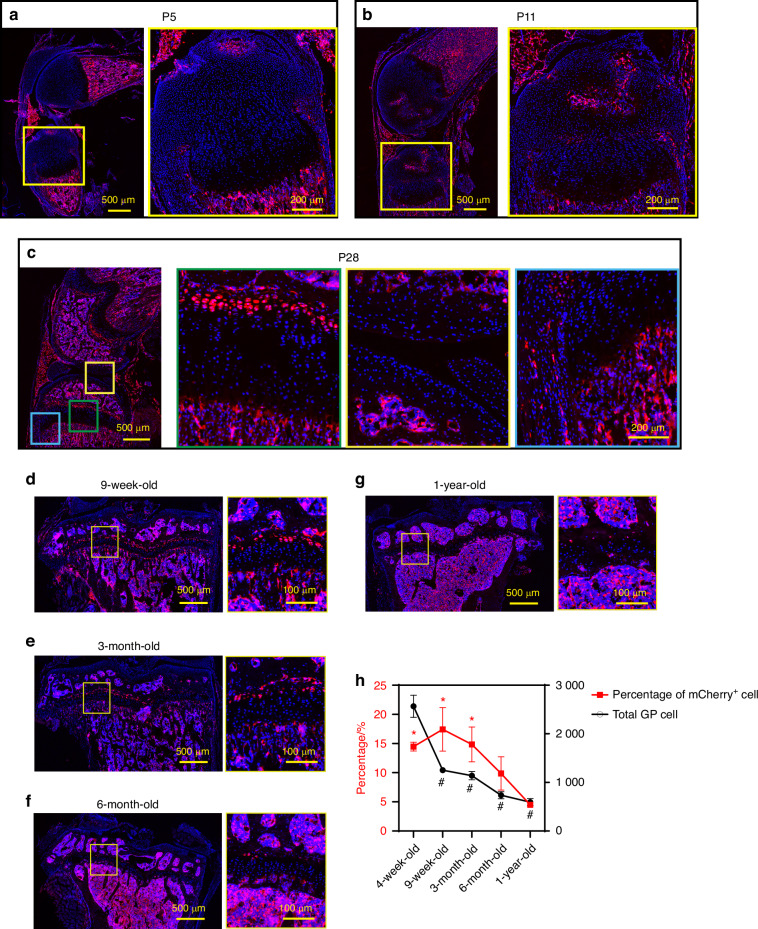
Histological analysis of *Apoe*^*mCherry/+*^ mice. **a**–**c** Sagittal sections of the left knees from *Apoe*^*mCherry/+*^ mice at postnatal day 5 (P5), day 11 (P11), and day 28 (P28, 4-week-old). **d**–**g** Fluorescence images of coronal sections of left tibiae from *Apoe*^*mCherry/+*^ mice at different ages as indicated. **h** Percentages of mCherry^+^ cells within the growth plate cells (red line) and the total number of growth plate chondrocytes (black line). 3-4 male *Apoe*^*mCherry/+*^ mice were used at each time point for quantification. Data are presented as mean ± SD. One-way ANOVA followed by Tukey’s multiple comparison test. *: *P* < 0.05 compared to the 1-year-old group (percentage of mCherry^+^ cell). #: *P* < 0.000 1 compared to the 4-week-old group (total GP cell)

To perform scRNAseq of growth plate chondrocytes, we harvested femora and tibiae from a female C57BL/6J mouse at 4 weeks of age during the growth spurt and removed as much soft tissue as possible under a stereomicroscope. The distal femoral epiphysis and proximal tibial epiphysis were then dislodged from the metaphysis (Fig. S1a). The dislodged epiphysis with the attached growth plate was then digested to isolate growth plate chondrocytes. Hematopoietic cells and red blood cells were removed by magnetic beads for CD45 and Ter119 antibodies, and the resulting single-cell suspension was subjected to scRNAseq on the 10x Genomics platform. Unsupervised data analysis using the Seurat package provided a uniform manifold approximation and projection (UMAP) with 11 distinct cell clusters based on their gene expression profiles (Fig. 1a). Based on the characteristics of the transcriptomes, these 11 clusters appeared to contain not only chondrocytes, but also other cell types such as osteoblasts, endothelial cells, ligamentous fibroblasts, and mesenchymal progenitor cells (Fig. 1b). To annotate the clusters with the goal of distinguishing growth plate chondrocytes from other cell types, we examined gene expression of marker genes that have been reported to be expressed in a specific cell type, followed by histologic validation by FISH on histological sections of 4-week old mice. Aggrecan (*Acan*) is a pan marker for chondrogenic cells, including articular chondrocytes (Fig. 1c-1, c-2), growth plate chondrocytes (Fig. 1c-1, c-3), and, at a lower expression level, perichondrial cells (Fig. 1c-1, c-4). To distinguish articular chondrocytes from growth plate chondrocytes, we used *Prg4* (proteoglycan 4 or lubricin).^17–19^ and *Epyc* (epiphycan).^20,21^ *Prg4* is highly expressed in articular chondrocytes and meniscal cells (Fig. 1d-1, d-2) as well as in ligament cells (Fig. 1d-3), but its expression in growth plate chondrocytes and perichondrial cells is minimal (Figs. 1d-4), consistent with a previous report.^22^ In contrast, *Epyc* is robustly expressed in growth plate chondrocytes with the highest expression in the columnar chondrocytes (Fig. 1e-1, e-3), but its expression level is much lower in articular chondrocytes (Fig. 1e-2) and perichondrial cells (Fig. 1e-4). Perichondrial cells specifically express *Postn* (periostin) (Fig. 1f). They also express relatively low levels of *Acan*, but higher levels of *Col1a1* compared to chondrocytes (Fig.1g). Notably, they can be distinguished from *Col1a1*-expressing osteoblasts, which are mainly located in the trabecular and endosteal areas, because they do not express *Bglap*, a marker for mature osteoblasts.^23^ Consistent with previous reports, a fraction of perichondrial/periosteal cells in the outer layer express *Ctsk*,^24–26^ although to a lesser extent compared to osteoclasts in the trabecular area (Fig. 1h). We also identified a cluster of cells expressing *Lepr* and *Pdgfrb*, two markers of bone marrow mesenchymal progenitor cells (MPCs).^27–29^ FISH confirmed that *Lepr*^+^*Pdgfrb*^+^ MPCs were mainly located in the trabecular region and not in the growth plate (Fig. 1i). In addition, we observed that these two markers, especially *Pdgfrb*, are robustly expressed in the perichondrium and periosteum (Fig. 1i), which is consistent with previous observations.^28,29^ With this scRNAseq analysis and FISH validation approach, we were able to annotate the 11 clusters with 5 clusters of growth plate chondrocytes (GP1-5, *Acan*^*high*^*Epyc*^+^*Prg4*^-^), articular chondrocytes (AC, *Acan*^*high*^*Prg4*^*+*^), perichondrial cells (PeriChon, *Postn*^*+*^*Acan*^*low*^*Col1a1*^*+*^*Bglap*^*-*^*Ctsk*^*+*^*Pdgfrb*^*+*^), ligamentous fibroblasts (LF, *Eln*^*+*^*Fn1*^*+*^*Prg4*^*+*^), osteoblasts (OB, *Col1a1*^*+*^*Bglap*^*+*^), mesenchymal progenitor cells (MPCs, *Pdgfrb*^*+*^*Lepr*^*+*^), and endothelial cells (EC, *Pecam1*^*+*^*Emcn*^*+*^) (Fig.1b).

### Apoe is ubiquitously and specifically expressed in growth plate RCs

To further analyze growth plate chondrocytes, clusters containing growth plate chondrocytes in Fig. 1a (GP1-5) were selected and re-clustered. The transcriptomic profiles of chondrocytes in different zones within the growth plate were analyzed and the clusters were annotated in a similar approach as in Fig. 1 (Fig. 2a, b). FISH confirmed the spatial gene expression of genes representing chondrocytes in each zone. For each gene, we compared the localization in tissue sections with our scRNAseq UMAP side-by-side to validate our annotation (Fig. 2c). *Clu* (Clusterin).^30^ and *Pthlh* (PTHrP),^11^ two previously reported markers for RCs, were specifically expressed in subsets of RCs in the upper layer of the growth plate (Fig. 2c, RC. Enlarged images for *Pthlh* are shown in Fig. S2a). *Ccnd1* (Cyclin D1), which modulates the transition from G1 to S phase,^31^ was mainly expressed at the boundary between unorganized RCs and column-forming proliferating chondrocytes (Fig. 2c, Mito-C, top), suggesting that *Ccnd1* labels chondrocytes entering the cell cycle from the quiescent state. On the other hand, the mitotic gene *Mki67*, which is maximally expressed in the G2 and M phases,^32^ was specifically expressed in a cluster in the UMAP, but these *Mki67*-expressing chondrocytes were sparsely distributed in the proliferative zone (Fig. 2c, Mito-C, bottom). *C1qtnf3* (CTRP3) was previously identified to be expressed in proliferating chondrocytes in the growth plate.^33^ Consistently, we confirmed that *C1qtnf3* was specifically expressed in the columnar chondrocytes such as proliferative and prehypertrophic chondrocytes (Fig. 2c, PC, top). In addition, glucose metabolism is an essential metabolic pathway in growth plate chondrocytes.^34–36^ *Pgk1*, encoding phosphoglycerate kinase 1, an essential glycolytic enzyme, was found to be highly expressed in the columnar chondrocytes compared to the RCs (Fig. 2c, PC, bottom). Finally, *Ihh* (Indian hedgehog) and *Col10a1* (Type X collagen) are robustly expressed in the prehypertrophic and hypertrophic chondrocytes, respectively (Fig. 2c, PHC/HC). Therefore, we annotated the clusters as shown in Fig. 2a. Pseudotime analysis of these five clusters also showed that the pseudotime score increased as chondrocytes shifted from RC to HC in the UMAP, which also supports our cluster annotation (Fig. 2d). After identifying the cluster for RCs, we searched for marker genes that were ubiquitously expressed in RCs (minimum percentage of expressing cells in the cluster > 90%) and specifically expressed in RCs (the minimum fold change compared to the other four clusters; logFC > 1). Among the 41 differentially expressed genes meeting these criteria, apolipoprotein E (*Apoe*) had the highest fold change (logFC = 3.87) and was expressed in the majority of cells in the RC cluster (>97%) (Fig. 2e). FISH confirmed that *Apoe*, *C1qtnf3*, and *Col10a1* could distinguish the RZ, PZ/PHZ, and HZ, respectively (Figs. 2f, S2b). Immunofluorescence staining with the APOE antibody revealed that APOE was highly expressed in chondrocytes in the RZ compared to the PZ of the human growth plate, suggesting that apolipoprotein E may be commonly expressed in RCs across species (Fig. 2g).

### Apoe^mCherry^ labels RCs after the formation of the secondary ossification center

To further characterize the *Apoe*-expressing RCs, we generated *Apoe*^*mCherry*^ reporter mice that express mCherry protein under the control of the endogenous *Apoe* gene promoter and enhancers (the mCherry-BGHpA cassette was inserted after the endogenous ATG start codon in exon 2 of the mouse *Apoe* gene) (Fig. 3a). This reporter model allows us to visualize the real-time *Apoe* expression in cells with mCherry to characterize *Apoe*^+^ growth plate chondrocytes in vivo and in vitro. Since this is a knock-in mouse model and there is only one allele of *Apoe* in the *Apoe*^*mCherry/+*^ mice, we first examined whether there is a haploinsufficiency for APOE that causes systemic or skeletal phenotypes. As shown in Fig. 3b, we did not find significant differences in body weight and femur length between *Apoe*^*+/+*^ and *Apoe*^*mCherry/+*^ mice. *Apoe* global knockout mice (*Apoe*^*-/-*^ mice) exhibit prominent hyperlipidemia,^37^ but we did not observe significant upregulation of triglyceride and cholesterol levels in 4-week-old *Apoe*^*mCherry/+*^ mice. Plasma APOE levels were decreased but did not reach a statistical significance. Lastly, we examined the growth plate histologically by FISH and found no significant alteration in the proportions of each zone (RZ, PZ, and HZ) in the *Apoe*^*mCherry/+*^ mice, although the length of *Apoe*^*+*^ RCs tended to decrease (Fig. 3c), possibly due to decreased *Apoe* expression. Collectively, these data indicate that the loss of one allele of *Apoe* in the *Apoe*^*mCherry/+*^ mice did not result in abnormal systemic or skeletal phenotypes.

Next, colocalization of mCherry protein and *Apoe* mRNA was confirmed by a combination of immunofluorescence and FISH (Fig. S3), indicating that mCherry expression is concomitant with endogenous *Apoe* expression. During postnatal skeletal development, mCherry expression was not detected in tibial epiphyseal cartilage at postnatal day 5 (P5), before SOC formation (Fig. 4a). At this stage, mCherry expression was primarily observed in the bone marrow, perichondrium, and part of the ligamentous cells. mCherry-expressing chondrocytes began to appear in the tibial RZ after SOC formation at P11 (Fig. 4b). At 4 weeks of age (P28), mCherry^+^ chondrocytes lined up in the RZ, but were not detected in other zones, while some expression was observed in the outer layer of the perichondrium and a few articular chondrocytes (Fig. 4c. the green, yellow, and blue boxes indicate the growth plate, the articular chondrocytes, and the perichondrium, respectively). These findings are consistent with our scRNAseq data of growth plate cells (Fig. 1a, b) and bone marrow cells (Fig. S4) showing that *Apoe* is highly expressed in endothelial cells, macrophages, and MPC, and at lower levels in perichondrial and articular chondrocytes. In later stages of life, the percentage of mCherry^+^ RCs in the growth plate peaks at 9 weeks of age and then decreases over time (Fig. 4d–h), suggesting that RCs in the growth plate are exhausted after skeletal maturity with aging, although a few mCherry^+^ RCs remain in the growth plate at 1 year of age.

### mCherry^+^ RCs are slow-cycling chondrocytes

**Fig. 5:**
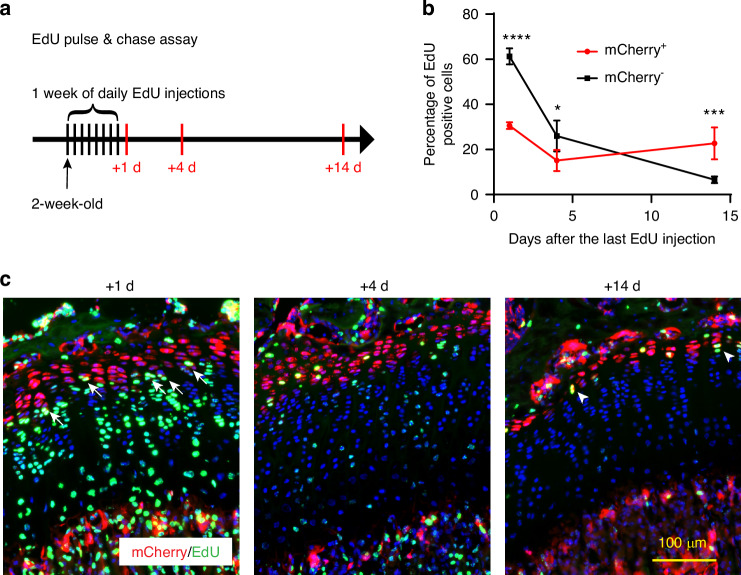
EdU pulse & chase assay in *Apoe*^*mCherry/+*^ mice. **a** Experimental scheme of the EdU injections and sampling. The 8-day EdU injection started at 2 weeks of age and tibiae were harvested 1, 4 and 14 days after the last injection. **b** Percentage of EdU^+^ cells in mCherry^+^ RCs (red) and mCherry^-^ chondrocytes (black) at the indicated time points. Data are from male mice and presented as mean ± SD. *n* = 3 independent animals for +1 d and +4 d and *n* = 4 for +14 d. *: *P* = 0.004, ***: *P* = 0.000 3, ****: *P* < 0.000 1. Two-way ANOVA (factor mCherry: *P* = 0.001 6; factor time: *P* < 0.000 1) followed by Tukey’s multiple comparison test. **c** Representative images of EdU detection (green) and mCherry immunofluorescence (red) with DAPI (blue). White arrows at +1 d indicate EdU^+^mCherry^+^ cells in the lower part of the RZ. White arrowheads at +14 d indicate two adjacent EdU^+^ nuclei, presumably divided from a single labeled nucleus

In contrast to proliferating columnar chondrocytes, “resting” chondrocytes are quiescent with a longer cell cycle.^38^ To determine whether mCherry^+^ chondrocytes are slow-cycling, we performed an EdU pulse-chase assay. Specifically, we injected EdU into 2-week-old *Apoe*^*mCherry/+*^ mice for 8 consecutive days and tracked EdU incorporation into growth plate chondrocytes at 1 day, 4 days and 14 days after the last injection (Fig. 5a). At this growing age, chondrocytes can pass through the growth plate within a few days.^16^ Thus, fast-cycling cells lose the EdU label in a few days after multiple cell divisions, while slow-cycling cells can retain it. One day after the 8-day injections, approximately 60% of proliferative chondrocytes (mCherry^-^) incorporated EdU, while only 30% of mCherry^+^ RCs had EdU, suggesting that fewer mCherry^+^ RCs entered the cell cycle during the 8 days compared to proliferative chondrocytes. Furthermore, as expected, proliferating columnar cells rapidly lost their EdU labeling down to ~30% by day 4 and ~6% by day 14 (Figs. 5b, c, S5a). In contrast, mCherry^+^ cells took much longer to lose their EdU labeling (~30% at day 1, ~15% at day 4, and ~22% at day 14) (Figs. 5b, c, S5a), indicating that mCherry^+^ RCs are slow-cycling compared to proliferative chondrocytes. Notably, we often found mCherry^+^EdU^+^ cells located in the lower RZ (white arrows in Fig. 5c, +1 d). Given the relatively high EdU incorporation rate (~30%) in the mCherry^+^ RCs, we reasoned that the mCherry^+^ RCs consisted of heterogeneous cell populations including the dormant stem cells in the upper RZ and activated progenitors, transitioning into proliferative chondrocytes in the lower RZ. Consistently, FISH analysis for *Apoe*, *Clu*, and *C1qtnf3*, revealed more than 30% overlap between *Apoe*^*+*^ RCs and *C1qtnf3*^*+*^ PCs at the boundary. On the other hand, *Clu* labeled the upper *Apoe*^*+*^ RCs and had little overlap with the *C1qtnf3*^*+*^ PCs (Fig. S5b), which was also confirmed by the feature plots for *Apoe*, *Clu*, and *C1qtnf3* in our scRNAseq data (Fig. S5c). Moreover, RCs in the lower RZ express *Ccnd1* as shown in Figs. 2b, c, and S5c, d, suggesting that these RCs are entering the cell cycle. The reduction in EdU positivity in the RCs from day 1 to day 4 might have derived from the transition of EdU^+^ RCs into the proliferative zone. The slight increase in EdU-positive cells in RCs from day 4 to day 14 may be due to cell division of the slow-cycling RCs in the upper RZ within the two-week window, as we observed some EdU-positive nuclei aligned close to each other, suggesting that they had just divided from a single nucleus (Fig.5c, +14 d, white arrowheads). These results suggest that our *Apoe*^*mCherry*^ reporter mice temporally and spatially label the RZ, which potentially consists of heterogeneous RCs.

### mCherry^+^ RCs are heterogenous and contain skeletal progenitor cells with high proliferative potential and multipotency in vitro

**Fig. 6:**
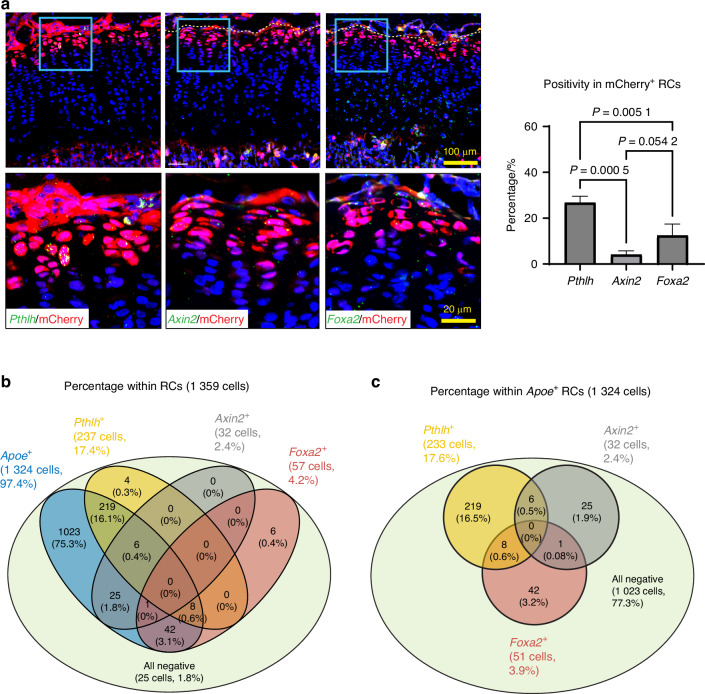
Heterogeneity of the mCherry^**+**^ RCs. **a** RNAscope of *Pthlh, Axin2*, and *Foxa2* on tibial sections from male *Apoe*^*mCherry/+*^ mice. The percentage of positive cells for each marker within mCherry^+^ RCs was quantified. Data are presented as mean ± SD. *n* = 3 independent animals. One-way ANOVA followed by Tukey’s multiple comparison test. **b** Colocalization analysis of *Apoe*^*+*^, *Pthlh*^*+*^, *Axin2*^*+*^ and *Foxa2*^*+*^ cells in the RC cluster of the scRNAseq data shown in Fig. 2a. **c** Colocalization analysis of *Pthlh*^*+*^, *Axin2*^*+*^ and *Foxa2*^*+*^ cells in the *Apoe*^*+*^ RCs of the scRNAseq data shown in Fig. 2a

After SOC formation, the RZ in the growth plate has been shown to harbor skeletal stem cells that express *Pthlh*,^11^ *Axin2*,^12,13^ or *Foxa2*.^14^ These skeletal stem cells supply chondrocytes that form columns in the growth plate during linear bone growth. Our histological evaluation by FISH found that 94% of the *Pthlh-*expressing stem cells expressed mCherry, but only 27% of the mCherry-expressing cells were *Pthlh* positive (Fig. 6a). On the other hand, the percentage of *Axin2*-expressing cells and *Foxa2*-expressing cells within mCherry^+^ cells were 4% and 12%, respectively (Fig. 6a), which was much lower than *Pthlh*^+^ cells. To gain a holistic understanding of these stem cell populations, we examined the positivity of *Apoe*, *Pthlh*, *Axin2*, and *Foxa2* gene expression in the RC cluster of our scRNAseq dataset shown in Fig. 2a. We considered positive cells when the raw count for each gene was greater than 0. Figure 6b shows that most RCs (97%) are *Apoe*^+^ (1 324 cells out of 1 359 cells), while *Pthlh*^+^, *Axin2*^+^ and *Foxa2*^+^ cells represent small subsets of RCs, 17% (237 cells out of 1 359 cells), 2% (32 cells out of 1 359 cells), and 4% (57 cells out of 1 359 cells), respectively. These percentages were slightly lower than what we observed in histology (Fig. 6a), possibly due to the different detection sensitivities between RNA sequencing and FISH. However, the proportion of these percentages among *Pthlh*^*+*^, *Axin2*^*+*^ and *Foxa2*^*+*^ cells is similar between scRNAseq analysis and FISH (17%, 2%, 4% in scRNAseq data, 27%, 4% and 12% in FISH, respectively). Based on our scRNAseq data, 77% of *Apoe*-expressing RCs (1 023 cells out of 1 324 cells) are negative for *Pthlh*, *Axin2*, and *Foxa2*. Interestingly, among the *Apoe*-expressing RCs that are positive for either *Pthlh*, *Axin2*, or *Foxa2*, over 95% of these cells (286 cells out of 301 cells) express only one of *Pthlh*, *Axin2*, or *Foxa2*, and there were no triple positive (*Pthlh*^*+*^*Axin2*^*+*^*Foxa2*^*+*^) cells in the RCs (Fig. 6c), suggesting that these genes label independent subsets of RCs and that skeletal stem cells in RCs are heterogeneous. Importantly, *Apoe* positivity in these subsets of *Pthlh*^+^, *Axin2*^+^ and *Foxa2*^+^ RCs is 98% (233 cells out of 237 cells), 100% (32 cells out of 32 cells), and 89% (51 cells out of 57 cells), respectively. These results suggest that *Apoe* is a common marker that spans the RCs, whereas *Pthlh*, *Axin2* and *Foxa2* exclusively label heterogeneous subsets of skeletal stem cells.

**Fig. 7:**
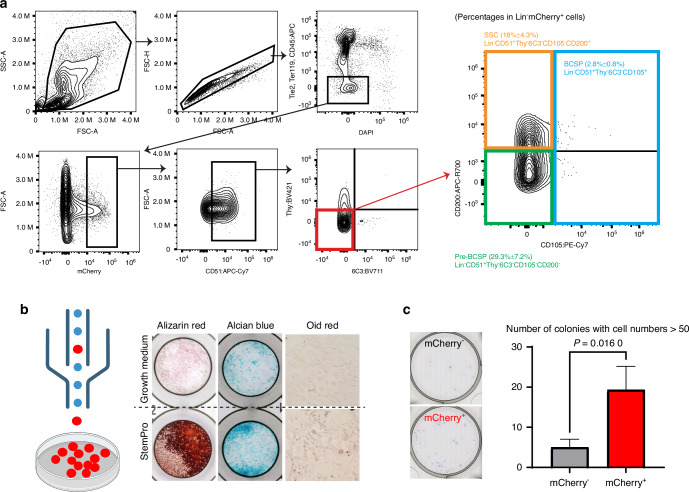
Characterization of mCherry^**+**^ RCs for stem cell properties. **a** Gating scheme for flow cytometric analysis of GP cells isolated from 4-week-old male *Apoe*^*mCherry/+*^ mice (*n* = 3 independent animals). Percentage is within DAPI^-^Lin^-^mCherry^+^ cells. Lin: hematopoietic and endothelial lineage cells (CD45^+^Tie2^+^Ter119^+^). **b** Lin^-^CD73^+^mCherry^+^ cells were sorted and subjected to in vitro osteogenic/chondrogenic/adipogenic differentiation assays. The upper panels are controls cultured in non-differentiating media. Representative figures from 3 independent experiments are shown. **c** Colony forming unit (CFU) assay comparing the self-renewal capabilities between Lin^-^CD73^+^mCherry^-^ and Lin^-^CD73^+^mCherry^+^ cells. Data are presented as mean ± SD. *n* = 3 replicates of pooled cells from 2 independent mice. Student’s *t*-test

Previous studies have identified a panel of mouse skeletal stem cell surface markers for flow cytometric analysis.^39^ We therefore examined the expression of these surface markers on mCherry^+^ RCs. Following our gating scheme to enrich for singlet cells, we removed dead cells and hematopoietic/endothelial cells labeled with CD45, Tie2, and Ter119. In the mCherry^+^ cells of the remaining population, we found that approximately half of the mCherry^+^ RCs had signatures of multipotent cells,^39^ of which ~18% had signatures of self-renewing mouse skeletal stem cells (SSC, CD45^-^Tie2^-^CD51^+^Thy^-^6C3^-^CD105^-^CD200^+^), ~ 29.3% had signatures of pre-bone, cartilage and stromal progenitors (pre-BCSP, CD45^-^Tie2^−^CD51^+^Thy^-^6C3^-^CD105^-^CD200^-^), and ~2.8% had signatures of bone, cartilage and stromal progenitors (BCSP, CD45^−^Tie2^−^CD51^+^Thy^−^6C3^−^CD105^+^) (Fig. 7a). These results demonstrated that mCherry^+^ RCs may contain skeletal stem cells as well as skeletal progenitor cells, further indicating the heterogeneity of RCs.

To functionally validate that mCherry^+^ cells have proliferative potential and multipotency as progenitor cells, we sorted mCherry^+^ cells for in vitro differentiation experiments. In addition to RCs, mCherry^+^ cells were also detected in the bone marrow (Fig. 4). Our scRNAseq analysis of bone marrow cells showed that *Apoe* was also expressed in endothelial cells, macrophages, and part of skeletal cells (Fig. S4). Therefore, to remove mCherry^+^ cells from the bone marrow and enrich mCherry^+^ RCs, in addition to CD31 and CD45, markers for endothelial cells and hematopoietic cells including macrophages, we utilized CD73, which has been reported as an RC marker.^9^ Previous studies have also shown that CD73 labels bone marrow cell fractions with high differentiation and proliferative potential, as well as the sinusoidal cells.^40,41^ To confirm that our sorting strategy can enrich mCherry^+^ cells from the growth plate but not from the bone marrow, we separately harvested cells from the growth plate and bone marrow of *Apoe*^*mCherry/+*^ mice and examined the positivity for CD73 and mCherry. The majority of mCherry^+^ cells isolated from the growth plate were positive for CD73 (> 90%), whereas only 1% of CD73^+^ non-hematopoietic cells from the bone marrow were mCherry^+^ (Fig. S6). Therefore, we sorted mCherry^+^ RCs as CD31^-^CD45^-^Ter119^-^CD73^+^mCherry^+^ cells and cultured them under osteogenic, chondrogenic, and adipogenic differentiation conditions (Fig. 7b). Although the contamination of bone marrow mCherry^+^CD73^+^ cells cannot be completely excluded, these cells should be quite minor compared to mCherry^+^CD73^+^ RCs because our chondrocyte isolation method contains a very small MPC population as shown in Fig. 1a. Therefore, this rare population is unlikely to affect our downstream in vitro analyses. The sorted mCherry^+^ RCs were able to differentiate into osteoblasts and chondrocytes as demonstrated by alizarin red and alcian blue staining (Fig. 7b). However, these mCherry^+^ RCs exhibited limited adipogenic differentiation potential (Fig. 7b), which is consistent with previous findings regarding the non-adipogenic skeletal stem cells.^39^ Lastly, we performed a colony-forming assay to determine whether mCherry^+^ RCs have higher proliferative potential than mCherry^-^ cells. When seeded at a low cell density (100 cells/cm^2^), mCherry^+^ RCs formed a significantly higher number of colonies with cell numbers > 50 compared to mCherry^-^ cells in vitro (Fig. 7c). These findings suggest that mCherry^+^ RCs contain skeletal progenitor cells that have high proliferative potential and multipotency toward osteo- and chondrogenesis in vitro.

## Discussion

Using scRNAseq analysis and FISH validation, we identified apolipoprotein E as a novel pan-marker for RCs of postnatal mouse limb growth plates. To our knowledge, this is the first study to take advantage of FISH to intensively correlate scRNAseq results with spatial information from histological sections of growth plates. Although scRNAseq analysis is a powerful tool for dissecting the transcriptomic profiles of heterogeneous skeletal cells, the need for digestion to obtain single-cell suspensions raises several concerns, including loss of spatial information, transcriptomic changes during digestion steps, inconsistency of cell isolation, and loss of rare cell populations. These hurdles may still prevent us from fully understanding the landscape of cell composition in skeletal tissues. For example, although several recent studies using scRNAseq-based approaches have identified novel skeletal stem cell populations,^39,42,43^ we still do not know the precise locations of these skeletal stem cells in the skeleton. Emerging spatial sequencing tools such as Visium or high-plex FISH such as Nanostring and Xenium may be better alternative approaches. However, technical challenges remain for hard tissues, resolution has not yet reached the single-cell level, and/or the number of genes in a panel is limited. Our approach, combining scRNAseq and FISH validation on tissue sections, allowed us to robustly examine the locations of cells expressing genes identified from scRNAseq analysis within the growth plate tissue, leading to the identification of appropriate cell clusters to be analyzed for comprehensive characterization (Fig. 2c, e, f).

Previously, laser capture microdissection has been employed to profile the transcriptomes of chondrocytes in different zones of the growth plate. When the resting zone was microdissected from the proximal tibial epiphyses of 1-week or 10-day-old rats, followed by microarray analysis, more than 40 genes were identified whose expression was significantly higher in chondrocytes in the RZ than in those in other zones.^15,44^ However, *Apoe* was not on the list, presumably because SOC formation was not complete at the age studied. On the other hand, when the transcriptomic profiles of RCs were compared between mice at postnatal day 2 and day 28, *Apoe* was one of the genes that were significantly upregulated at day 28, whereas its expression was minimal at day 2.^9^ These previous findings are consistent with our results that RCs begin to express *Apoe* after SOC formation.

Several different genes have been used to mark stem cells in the RZ, such as *Pthlh*, *Axin2* and *Foxa2*.^11–14^ Lineage tracing studies of these genes have demonstrated that stem cells expressing these genes can form columns in the growth plate during longitudinal bone growth. There may be a single type of stem cell in the RZ, and these genes may mark the same stem cells at different stages of development or differentiation. However, the lineage tracing studies found that none of these genes can label the entire growth plate columns or the entire RCs, suggesting that the RCs are heterogeneous and that there may be other stem cell populations in the RZ. Indeed, as shown in Fig. 6b, c, our scRNAseq analysis revealed that there was little overlap in the expression of these genes in RCs, and no cell expressed all 3 genes simultaneously, also suggesting that several different types of stem cells exist in RCs. This heterogeneity of stem cells in the RZ is further supported by some observations in previous studies. For example, the dependence of canonical Wnt signaling appears to be opposite between *Axin2*^*+*^ cells^12^ and *Pthlh*^+^ cells,^22^ and *Foxa2*^+^ cells and *Pthlh*^+^ cells are geographically separated and primarily localized in the upper and lower layers of the RZ, respectively.^14^ Further investigation is required to dissect the cell composition of RCs. Given that the majority of cells expressing *Pthlh*, *Axin2*, or *Foxa2* are included in mCherry^+^ RCs in our *Apoe*^*mCherry/+*^ mice, this mouse model may be useful for studies to comprehensively characterize the heterogeneous RC population.

*Apoe* labels RCs in the growth plate after SOC formation (Fig. 4). Thus, these RCs must supply chondrocytes to the growth plate during postnatal growth. Compared to the embryonic and neonatal stages before SOC formation, postnatal growth has a slower growth rate.^45^ However, the total amount of growth is much greater and extends over a longer period of time. During this period, individuals are exposed to external stimuli from nutritional status, injury, and disease that can significantly affect skeletal growth. In these situations, RCs play an essential role by sensing systemic cues such as nutrient deprivation,^13^ modulation of growth plate activity,^46^ or regeneration.^14^ The *Apoe*^*mCherry/+*^ mice generated in this study would be a useful tool to further characterize RCs under these conditions and to understand the mechanisms underlying skeletal growth retardation.

Interestingly, *Apoe*-positive cells were still present in the growth plate even at 1 year of age, although the number was significantly reduced (Fig. 4g-h). In contrast to humans, mouse long bone growth plates do not close, suggesting that mouse growth plates are still equipped with the necessary cellular components, including stem cells, even after their skeletal maturity and may retain the potential to grow further if they are in the growing environment. Therefore, it would be interesting to investigate the differences between mCherry^+^ RCs in young and old (after growth arrest) to understand the mechanism underlying growth plate inactivation during aging.

We have demonstrated that apolipoprotein E is highly and specifically expressed in RCs in both mouse and human growth plates. This conserved expression of apolipoprotein E across species may suggest that APOE plays a biological role in the growth plate. However, it remains unknown whether APOE expression has any biological significance. It has been reported that mice with global deletion of *Apoe* (*Apoe*^-/-^ mice) have significantly shorter nose-rump lengths compared to wild-type mice.^47^ We also found that *Apoe*^-/-^ mice had significantly lower body weights and shorter femora compared to their wild-type littermates (Fig. S7). These findings suggest that *Apoe* may not only be a marker of RCs but may also have a functional role in longitudinal bone growth. Exogenous APOE is known to bind to lipids and facilitate their intercellular transport. Additionally, studies have shown that endogenous APOE plays a different role and contributes to intrinsic lipid metabolism in neural stem cells.^48–50^ It is thus plausible that the endogenous APOE is also involved in lipid metabolism in RCs. Although the current consensus is that lipid metabolism in the growth plate is minimal due to the absence of vascularization,^51^ the fact that *Apoe*-expressing RCs were present only after the formation of the SOC, a well-vascularized structure that is in close proximity to and potentially interacts with RCs, may imply an overlooked metabolic shift in these cells to acquire additional potentials to facilitate postnatal growth.^9^ The role of APOE in RCs is currently being investigated in our laboratory. Once identified, it will help us address the question of why *Apoe*-expressing RCs emerge after SOC formation.

In conclusion, using scRNAseq analysis and the FISH validation approach, we have successfully identified *Apoe* as a specific and ubiquitous marker of RCs in the mouse growth plate. APOE expression was also detected in human growth plate RCs. Using the newly generated *Apoe*^*mCherry*^ mice, *Apoe*-expressing RCs were shown to contain skeletal stem cells, consistent with RC characteristics. Therefore, *Apoe* is the first pan marker to label RCs and the *Apoe*^*mCherry*^ model would be a useful tool to further investigate RC biology and function.

## Materials and methods

### Mice

The University of Maryland School of Medicine IACUC has reviewed and approved all the mouse experiments in this study (IACUC protocol #0121007 and #00000649). C57BL/6 J mice were obtained from The Jackson Laboratory (mouse #000664). *Apoe*^*mCherry/+*^ mice were generated by the Ingenious Targeting Laboratory (Ingenious Targeting Laboratory, USA) using the following strategy. The targeting vector was designed to insert the mCherry-BGHpA cassette with a selection marker FRT-flanked Neo right after the ATG start codon of the *Apoe* gene in exon 2. This targeting vector contains ~6.1 kb of long homology arm at the 5’ end and ~2 kb of short homology arm at the 3’ end. 10 µg of the targeting vector was linearized and then transfected into FLP 129/SvEv x C57BL/6 hybrid embryonic stem cells (ES cells) by electroporation. After selection with the antibiotic G418, surviving clones were expanded for PCR analysis to identify recombinant ES clones. The Neo cassette in the targeting vector was removed during ES clone expansion. Once confirmed, the recombinant ES cells were microinjected into CD-1 blastocysts, and the resulting chimeras with a high percentage of agouti coat color were mated to C57BL/6 N wild-type mice to generate the F1 generation. Neo deletion, FLP transgene presence, long arm junction, and short arm integration were confirmed by PCR. The confirmed F1 mice were then backcrossed to C57BL/6 J wild-type mice for at least 7 generations. *Apoe*^*mCherry/+*^ mice were crossed with C57BL/6 J wild-type to produce *Apoe*^*mCherry/+*^ mice. Mice were genotyped by PCR using the following primers. A forward primer for the *mCherry* sequence 5’-GTCCGTGTCGCGAAGTTCCTATACTTTC-3’, a forward primer for the wildtype genome 5’-TGGCCCTCTGTGAAGTGGAATTTGAAC-3’, and a common reverse primer 5’-GAGGTCAGAGTTCTAGAGGGAATTGG-3’ were used. Both male and female mice were used in this study, and at least 3 mice per sex were used for quantification in each experiment.

### Human growth plate

Growth plate tissue was obtained from the radius of a de-identified adolescent male patient during epiphysiodesis surgery. The tissues were fixed in 10% formalin for 7 days and embedded in the paraffin blocks. The University of Maryland IRB determined that this project met the definition of “Not Human Subject Research” (HM-HP-00079767-1).

### Single-cell RNA-seq and data analysis

Chondrocytes were isolated from the growth plate according to a previously described protocol with some modifications.^22^ Briefly, after careful removal of soft tissues including the perichondrium, the epiphyses in the distal femur and proximal tibia were dislodged from the metaphyseal end at the osteochondral junction just below the growth plate (Fig. S1). The growth plates attached to the epiphyses were then digested in HBSS (Ca^-^Mg^-^) with 2 units of Liberase TM (#5401119001, Sigma) at 37 °C for 3 cycles of 30-minute incubations with agitation. We divided the digestion period into three cycles and washed the released cells with HBSS in each cycle to minimize the potential transcriptomic alterations due to prolonged digestion. Released cells were mechanically disassociated into a single-cell suspension by gently passing through an 18-gauge needle on a 10 mL syringe 5-10 times, followed by filtering through a 70-µm cell strainer. Hematopoietic cells (CD45^+^, #103105, BioLegend) and red blood cells (Ter119^+^, #116207, BioLegend) were excluded by magnetic beads (BLD-480071, BioLegend) using the MACS manual separator (Miltenyi Biotec). Cell concentration (1 840 000 cells/mL) and viability (96%) were quantified and considered suitable for downstream analysis (cell viability > 70%). After encapsulation into emulsion droplets in the Chromium Controller (10× Genomics), libraries were constructed using 3’ GEM Library Prep (10× Genomics). cDNA libraries were profiled on a NovaSeq6000 sequencer using 100-cycle paired-end reads, targeting 10 000 cells per sample and 50 000 reads per cell. Data were processed using the 10× Genomics workflow. Cell Ranger (10× Genomics) was used for demultiplexing, barcode assignment, and unique molecular identifier (UMI) quantification. Downstream analysis was performed using Seurat v5.1. Cells with >7 500 and <1 000 expressed genes and >5% mitochondrial transcripts were excluded. Data were normalized using the SCTransform normalization method before principal component analysis and UMAP. Pseudotime analysis was performed using the Monocle 3 package in R.

### EdU labeling and detection

For 5-ethynyl-2’-deoxyuridine (EdU) pulse-chase experiments, 10 µg/g of EdU per mouse body weight (INV-A10044, Invitrogen) was injected peritoneally at the indicated time points. After permeabilization with 0.5% Triton X-100 (#9002-93-1, Sigma-Aldrich) in PBS for 30 min at room temperature, the Click-iT EdU Alexa Fluor 647 or Alexa Fluor 488 Imaging Kit (C10340 or C10329, Thermo Fisher) was used to detect EdU. The entire proximal tibial growth plate was used for analysis. The percentage of EdU^+^ cells in mCherry^+^ cells or that in mCherry^-^ chondrocytes in the entire growth plate was measured using Qupath.^52^

### RNA in situ hybridization (RNAScope)

RNAScope was performed on tibiae as previously described.^16,53^ Briefly, 2% formaldehyde-fixed, undecalcified bones were embedded in the OCT compound and the frozen blocks were sectioned at 10 µm using Kawamoto film.^54^ The films were fixed with grease on glass slides with the sections facing up for downstream procedures. Endogenous peroxidase was blocked with 0.3% hydroxyperoxide, tissue permeabilization was performed by using Protease III, and RNAScope probe hybridization was performed using the RNAscope Multiplex Fluorescent V2 Assay kit according to the manufacturer’s protocol (Advance Cell Diagnostics Inc., Newark, CA, USA). The following probes were used in this study: Apoe (#313271), Clu (#427891), Pthrp (#456521), Prg4 (#437661), C1qtnf3 (#531451), Ctsk (#464071), Epyc (#572901), Pgk1 (#312961), Slc2a1 (#458671), Col1a1(#319371), Bglap (#478941), Lepr (#402731), Pdgfrb (#411381), Mki67 (#416771), Ccnd1 (#442671), Ihh (#413091), and Col10a1 (#467961). Stained images were captured with a BZ-X All-in-One Fluorescence Microscope (Keyence) or a CSU-W1 spinning disk confocal microscope (Nikon).

### Immunofluorescence staining

2% formaldehyde- or 4% paraformaldehyde-fixed cryo-sections were stained with primary antibodies against mCherry (1:50, INV-M11217, Invitrogen). Human paraffin sections were stained with human APOE (1:100, PA5-27088, Invitrogen) for 1 h at room temperature. For mCherry staining, antigen retrieval was performed by 0.25% trypsin for 10 min at room temperature prior to reaction with the primary antibody. For APOE staining, antigen retrieval was performed using pH 6.0 citrate buffer at 90 °C for 10 min. Sections were then incubated with anti-rat (1:200, A21434, Invitrogen) or anti-rabbit (1:200, A31572, Invitrogen) secondary antibodies for 1 h at room temperature.

### Blood tests

At 4 weeks of age, mice were sacrificed, blood samples were collected by cardiac puncture, and femurs were harvested for micro-CT scanning. Plasma triglyceride (#10010303, Cayman Chemical), total cholesterol (J3190, Promega), and APOE (CUSABIO) levels in mice were measured using colorimetric assay kits and an ELISA kit, respectively, following the manufacturer’s protocols.

### Micro CT analysis

Femurs were fixed in 4% PFA for 3–5 days and scanned in PBS with SkyScan 1172 (Bruker) at a 0.6° increment angle with averaging 2 frames using a 55 kV/181 μA x-ray source with a 0.5 mm Al filter and a beam flattener to reduce beam hardening artifacts. Images were reconstructed at a 10 μm isotropic voxel size for bone length measurement.

### Flow cytometry and cell sorting

Both hindlimbs were harvested from 4-week-old *Apoe*^*mCherry/+*^ mice. Growth plate chondrocytes were harvested as described for the single-cell RNA-seq and data analysis. Single cells were stained with the following cell surface markers and subjected to flow cytometrical analysis or cell sorting using an Aurora 4 UV or an Aurora CS (Cytek Biosciences): CD45-APC (#103112, BioLegend), CD45-BV421 (#103134, BioLegend), CD31-BV421 (#102424, BioLegend), Tie2-APC (#124010, BioLegend), Ter119-APC (#116212, BioLegend), Ter119-BV421 (#116234, BioLegend), CD73-PE/Cy7 (#127223, BioLegend), CD90.1 (#202529, BioLegend), CD90.2 (#105341, BioLegend), CD51-biotin (#104104, BioLegend), Ly-51-BV711 (#740691, BD Biosciences), CD105-PE/Cy7 (#120410, BioLegend), and CD200-APC/R700 (#565546, BD Biosciences). CD51 antibody was biotinylated and detected by streptavidin conjugated with APC/Cy7 (#405208, BioLegend). To label endothelial cells, Tie2-APC was used for SSC analysis in Fig. 6a and CD31-BV421 was used for sorting.

### In vitro cell differentiation assays and colony forming unit assay

Sorted CD73^+^mCherry^+^CD45^-^CD31^-^Ter119^-^ chondrocytes were seeded on 96-well culture plates at a density of ~20 000 cells/cm^2^. Osteogenic, chondrogenic, and adipogenic differentiation were induced using StemPro Differentiation Kits (A1007201/A1007001/A1007101, ThermoFisher). Alizarin Red S staining (A5533-25G, Sigma) was performed on day 7 after osteogenic induction to assess mineralization. Alcian blue (J60122, ThermoFisher) or Oil red O (O0625-25G, Sigma) staining was performed on day 14 to assess proteoglycan synthesis and lipid droplet formation as indicators of chondrogenic and adipogenic differentiation, respectively. DMEM supplemented with 10% FBS was used as control. For the CFU-F assay, sorted cells were seeded on 6-well plates at a density of 100 cells/cm^2^ and cultured in DMEM supplemented with 20% FBS for 7 days. Colonies were stained with crystal violet (C6158-50G, Sigma).

## Supplementary information

Supplementary information

## Data Availability

All data are presented in this manuscript. ScRNAseq dataset generated in this study has been deposited in the Gene Expression Omnibus (GEO) under accession number GSE288529. Data that are mentioned but not shown in this manuscript are available from the corresponding author upon reasonable request.
