# Supplementary material for: Apolipoprotein E is a marker of all chondrocytes in the growth plate resting zone
Source: Bone Res. 2025 Mar 3;13:31. doi: 10.1038/s41413-025-00407-2 (PMC11873292; doi:10.1038/s41413-025-00407-2)
Supplement: Supplementary file 1 — Supplementary information [file 41413_2025_407_MOESM1_ESM.pdf]

Supplementary information

Apolipoprotein E is a marker of all chondrocytes in the growth plate resting zone.

Joe Kodama, Takeshi Oichi, Kevin J. Wilkinson, Joshua M. Abzug, Takashi Kaito, Motomi Enomoto-Iwamoto, Masahiro Iwamoto, Satoru Otsuru

Correspondence:

Satoru Otsuru, MD, PhD

Associate Professor

Department of Orthopaedics

University of Maryland School of Medicine

670 W Baltimore Street, HSFIII Room 7173

Baltimore, MD 21201

Tel: 410-706-2048

sotsuru@som.umaryland.edu

This file includes:

Supplementary figures and legends S1 to S7

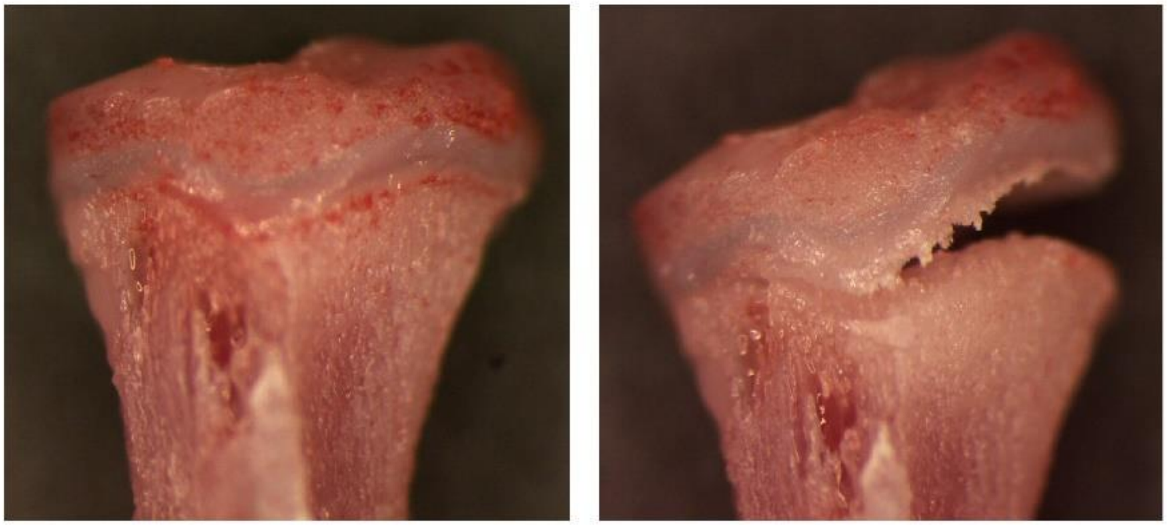

Figure S1. Photographs of the tibia before and after dislodging prior to digestion. Epiphyses along with the growth plates were dislodged from the metaphysis and subjected to digestion.

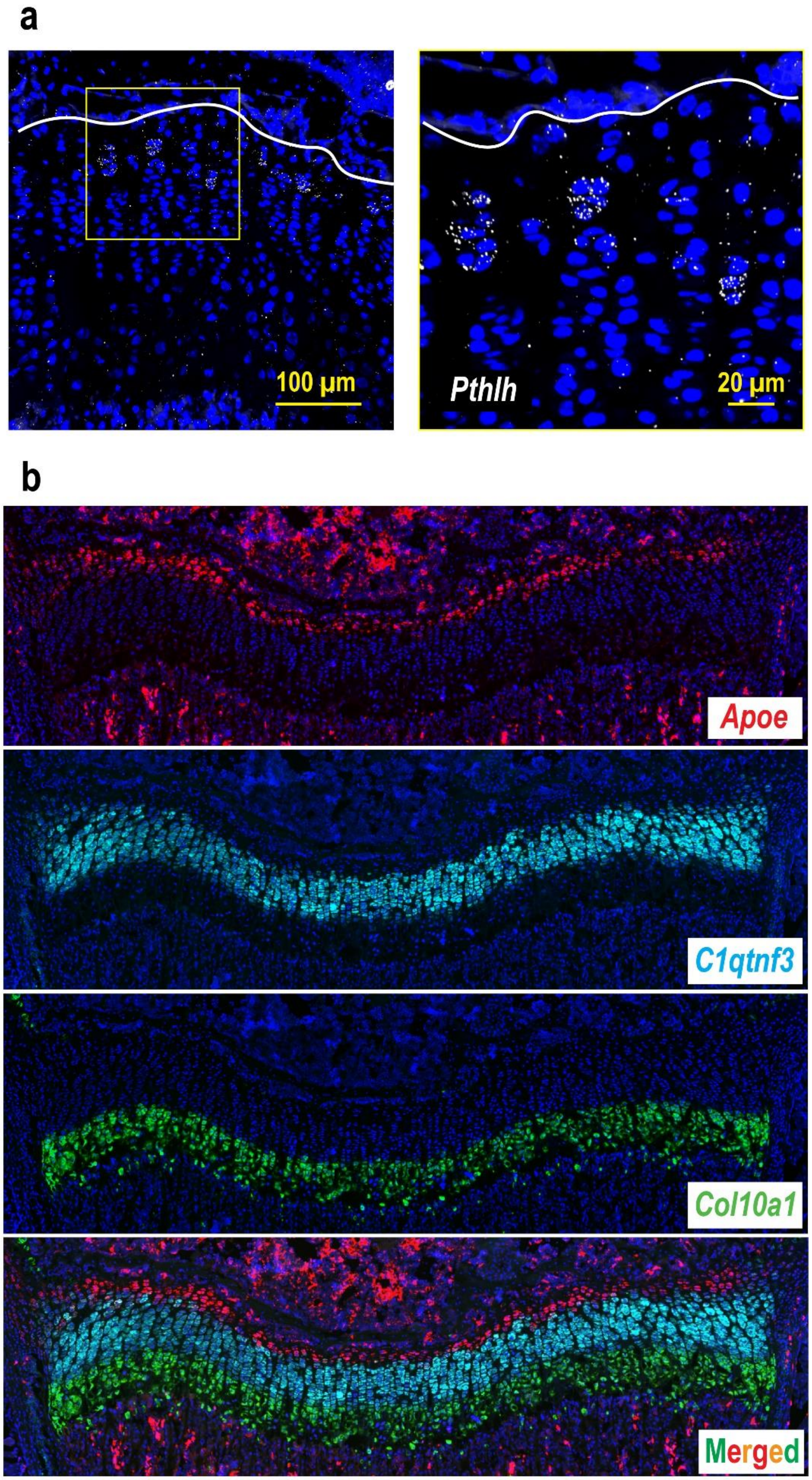

24

Figure S2. (a) FISH (RNAscope) for *Pthlh*. White lines indicate the boundary between the growth plate and the secondary ossification center. (b) FISH for *Apoe* (red), *C1qtnf3* (light blue), and *Col10a1* (green) with DAPI (blue) on a coronal tibia section from a 4-week-old male C57BL/6J mouse. Representative images from >3 animals.

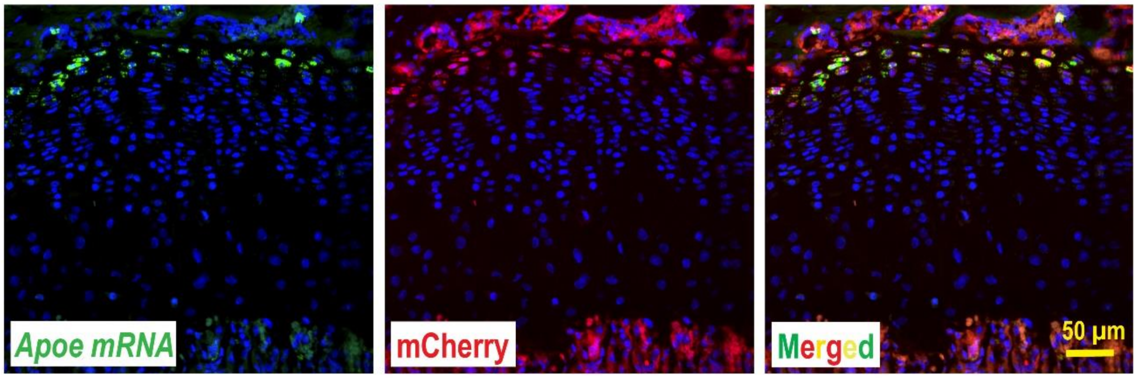

Figure S3. Co-staining of *Apoe* (mRNA by FISH) and mCherry (protein by immunofluorescence) on a coronal tibia section of a 4-week-old male *Apoe*$^{mCherry/+}$ mouse. Representative images from >3 animals.

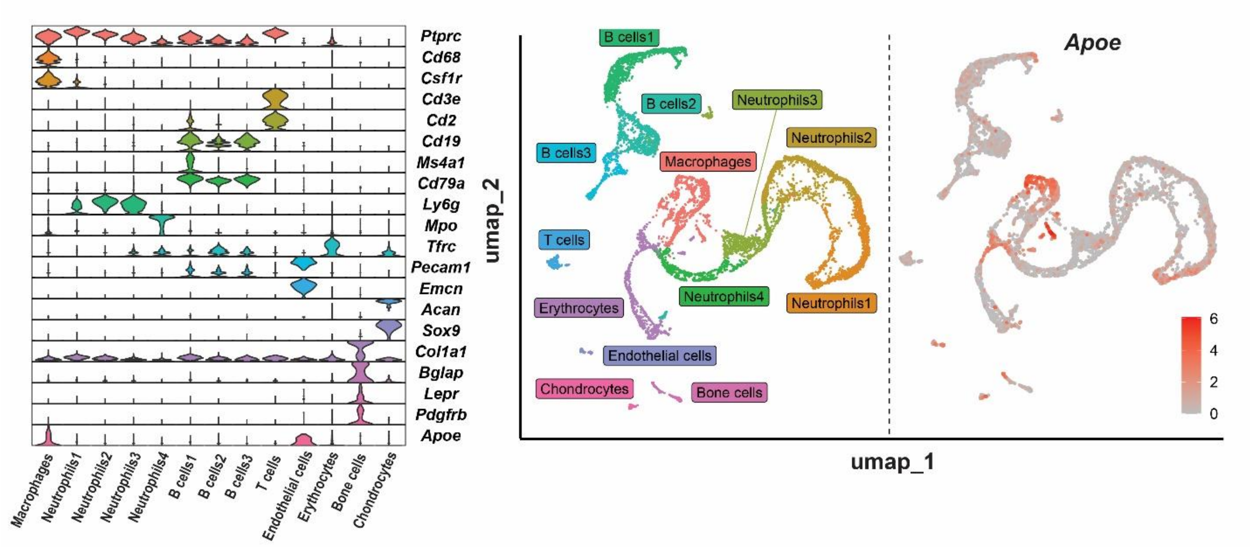

Figure S4. ScRNAseq of bone marrow cells from a 3-week-old male C57BL/6J mouse. Bone marrow was flushed out into a collection tube and the empty bones were digested in collagenase I solution (2.5 mg/mL) for 30 minutes twice (1 hour total) to isolate bone cells. Bone marrow fraction and the digested bone cell solution were mixed at 1:10. cDNA libraries were profiled on a NovaSeq6000 sequencer using 100-cycle paired-end reads, targeting 10,000 cells per sample and 50,000 reads per cell. Data were processed using the 10× Genomics workflow. Cell Ranger (10× Genomics) was used for demultiplexing, barcode assignment, and unique molecular identifier (UMI) quantification. Downstream analysis was performed using Seurat v5.1. Cells with >7,500 and <1000 expressed genes and >5% mitochondrial transcripts were excluded. Data were normalized using the SCTransform normalization method before principal component analysis and UMAP.

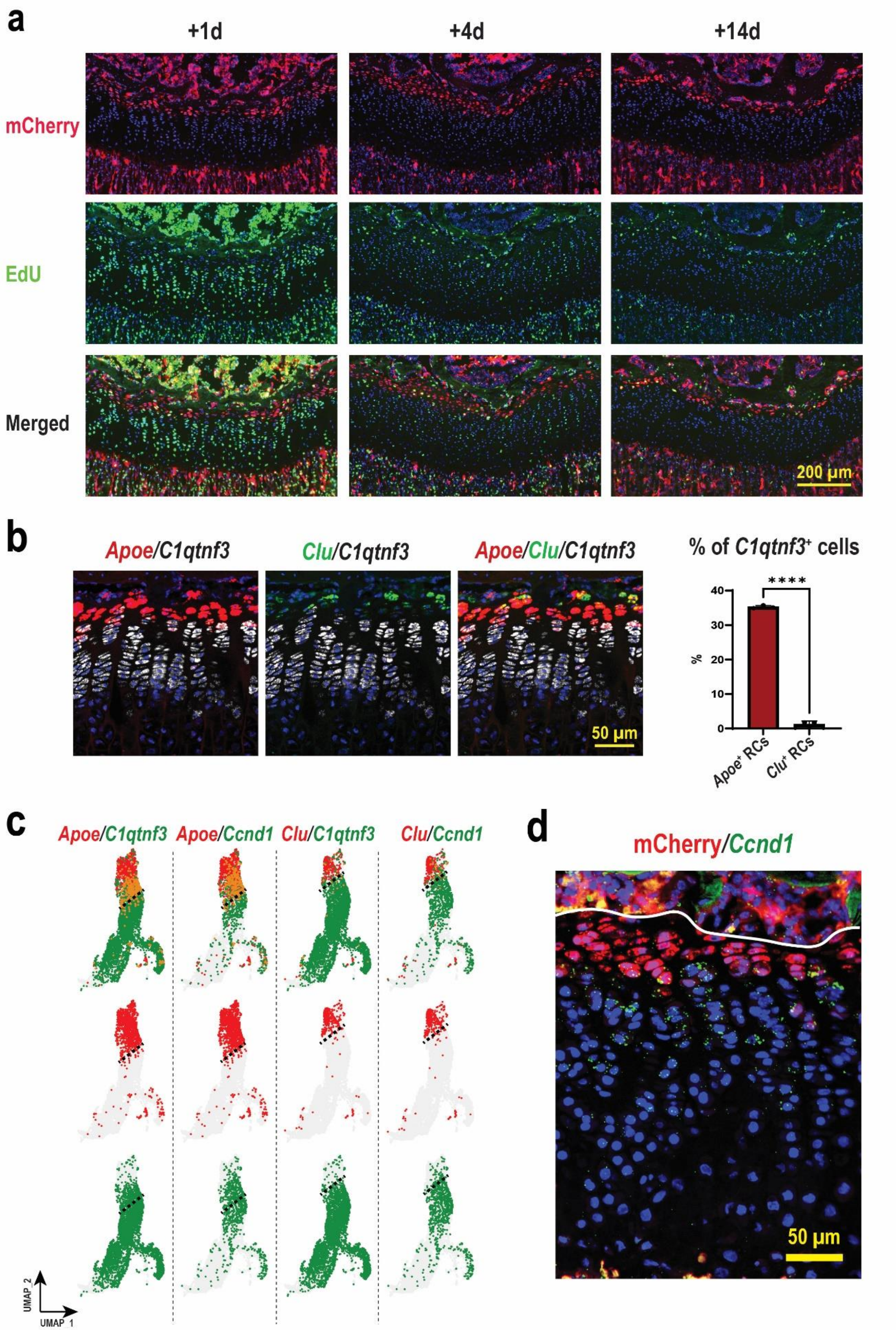
a
+1d
+4d
+14d
mCherry
EdU
Merged
200 µm
b
Apoe/C1qtnf3
Clu/C1qtnf3
Apoe/Clu/C1qtnf3
50 µm
% of C1qtnf3+ cells
****
%
40
30
20
10
0
Apoe+ RCs
Clu+ RCs
c
Apoe/C1qtnf3
Apoe/Ccnd1
Clu/C1qtnf3
Clu/Ccnd1
UMAP 2
UMAP 1
d
mCherry/Ccnd1
50 µm

Figure S5. (a) Representative images of EdU staining on tibia sections from *Apoe*$^{mCherry/+}$ male mice 1, 4, or 14 days after the last EdU injection (n=3~4 independent animals). (b) Colocalization between *Apoe*$^{+}$ or *Clu*$^{+}$ cells and *C1qtnf3*$^{+}$ cells. FISH was performed on sections from 3 independent 4-week-old wild-type male C57BL/6J mice. Data are presented as mean ± SD. ****: $p < 0.0001$. Student’s t-test. (c) Two genes (*Apoe* with *C1qtnf3, Apoe* with *Ccnd1, Clu* with *C1qtnf3,* or *Clu* with *Ccnd1*) are visualized in the same Feature plot (Seurat function, "Blend = TRUE"). Dashed lines were manually drawn to estimate the bottom line of *Apoe*-expressing or *Clu*-expressing RCs within the UMAP plot. (d) FISH for *Ccnd1* on a tibia section from a 4-week-old male *Apoe*$^{mCherry/+}$ mouse. White line indicates the boundary between the growth plate and secondary ossification center. A representative image from >3 animals.

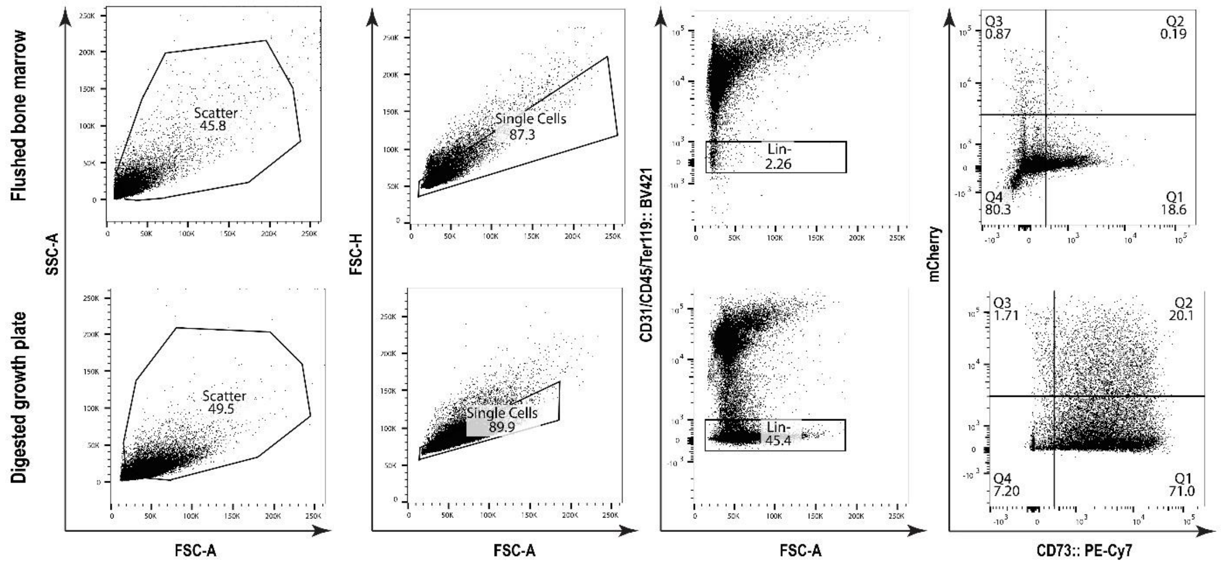

Figure S6. Flow cytometric analysis of flushed bone marrow cells and digested growth plate cells shows that $Lin^{-}mCherry^{+}$ cells in the bone marrow are predominantly $CD73^{-}$, whereas Lin^{-} $mCherry^{+}$ cells isolated from the growth plate are mostly $CD73^{+}$ (n=3 independent animals).

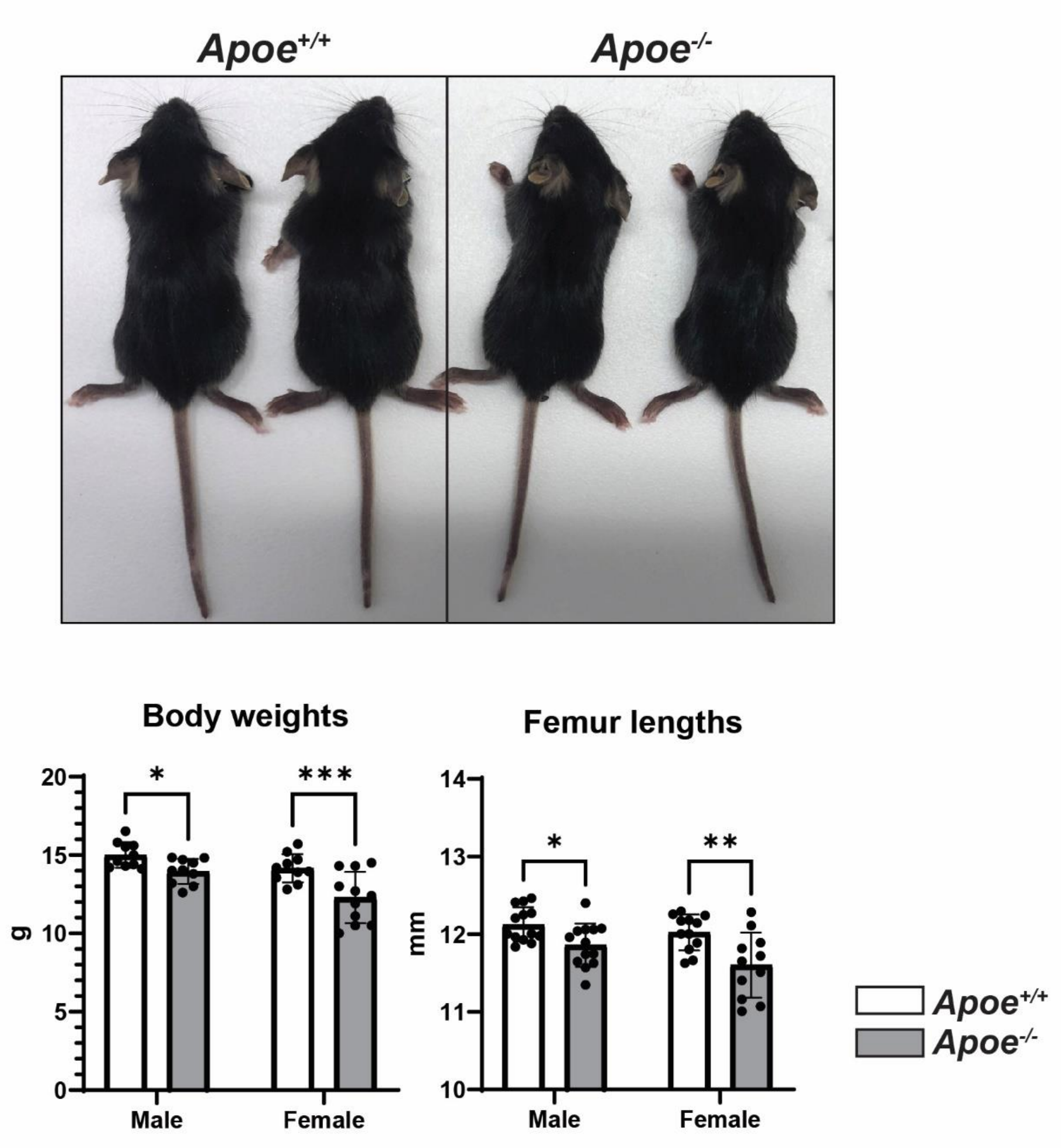

Figure S7. *Apoe* global knockout mice (Apoe$^{-/-}$ mice) are smaller than their wild type littermates (*Apoe*$^{+/+}$ mice). The top image shows the appearance of 4-week-old male *Apoe*$^{+/+}$ and *Apoe*$^{-/-}$ mice. Body weights (*Apoe*$^{+/+}$ males = 10, *Apoe*$^{+/+}$ females = 10, *Apoe*$^{-/-}$ males = 10, *Apoe*$^{-/-}$ females = 11) were scaled and femur lengths were measured by micro-CT (*Apoe*$^{+/+}$ males = 13, *Apoe*$^{+/+}$ females = 12, *Apoe*$^{-/-}$ males = 13, *Apoe*$^{-/-}$ females = 11) at 4 weeks of age. Samples were collected from independent animals. Data are presented as mean ± SD. *: p< 0.05, **: p< 0.01, ***: p<0.001. Two-way ANOVA followed by uncorrected Fisher's LSD test.
